# Assembling a Coculture System to Prepare Highly Pure Induced Pluripotent Stem Cell-Derived Neurons at Late Maturation Stages

**DOI:** 10.1523/ENEURO.0165-24.2024

**Published:** 2024-07-23

**Authors:** Masuma Akter, Masood Sepehrimanesh, Wu Xu, Baojin Ding

**Affiliations:** ^1^Department of Biochemistry and Molecular Biology, Louisiana State University Health Sciences Center at Shreveport, Shreveport Louisiana 71130-3932; ^2^Department of Chemistry, University of Louisiana at Lafayette, Lafayette Louisiana 70504

**Keywords:** coculture, human induced pluripotent stem cells (hiPSCs), motor neurons (MNs), neurodevelopment, synaptogenesis, transcriptomics

## Abstract

Generation of human induced pluripotent stem cell (hiPSC)-derived motor neurons (MNs) offers an unprecedented approach to modeling movement disorders such as dystonia and amyotrophic lateral sclerosis. However, achieving survival poses a significant challenge when culturing induced MNs, especially when aiming to reach late maturation stages. Utilizing hiPSC-derived motor neurons and primary mouse astrocytes, we assembled two types of coculture systems: direct coculturing of neurons with astrocytes and indirect coculture using culture inserts that physically separate neurons and astrocytes. Both systems significantly enhance neuron survival. Compared with these two systems, no significant differences in neurodevelopment, maturation, and survival within 3 weeks, allowing to prepare neurons at maturation stages. Using the indirect coculture system, we obtained highly pure MNs at the late mature stage from hiPSCs. Transcriptomic studies of hiPSC-derived MNs showed a typical neurodevelopmental switch in gene expression from the early immature stage to late maturation stages. Mature genes associated with neurodevelopment and synaptogenesis are highly enriched in MNs at late stages, demonstrating that these neurons achieve maturation. This study introduces a novel tool for the preparation of highly pure hiPSC-derived neurons, enabling the determination of neurological disease pathogenesis in neurons at late disease onset stages through biochemical approaches, which typically necessitate highly pure neurons. This advancement is particularly significant in modeling age-related neurodegeneration.

## Graphical abstract

**Figure:**
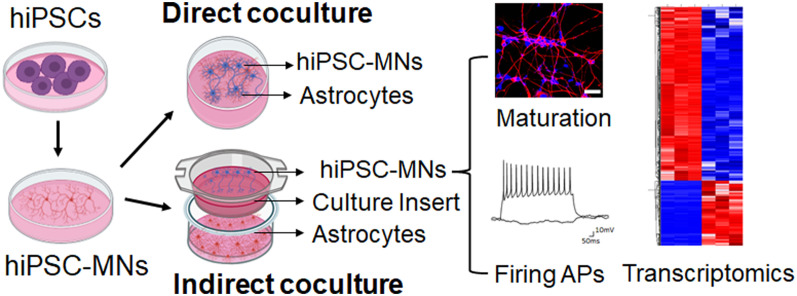
Visual Abstract

## Significance Statement

Achieving survival poses a significant challenge for long-term neural cell cultures. Utilizing hiPSC-derived motor neurons and primary mouse astrocytes, we established an indirect coculture system using culture inserts that physically separate neurons and astrocytes, thereby facilitating neuronal maturation. Transcriptomic studies revealed the typical neurodevelopmental switch in gene expression from the early immature stage to late maturation stages, indicating the high quality and maturation of neurons prepared with culture inserts. This study introduces a novel tool for the preparation of highly pure hiPSC-derived neurons, enabling the determination of neurological disease pathogenesis in neurons at late disease onset stages through biochemical approaches, which typically necessitate highly pure neurons. This advancement is particularly significant in modeling age-related neurodegeneration.

## Introduction

Generation of human induced pluripotent stem cell (hiPSC)-derived motor neurons (MNs) provides an unprecedented approach in the treatment of spinal cord injury (Saijo et al., 2024) or modeling movement disorders such as dystonia and amyotrophic lateral sclerosis (Akter and Ding, 2022; Ding, 2022; Giacomelli et al., 2022). However, the survival issue of induced MNs poses a significant challenge and limits its potential application in research, especially when aiming to prepare neurons at late maturation stages. Another challenge in modeling movement disorders using hiPSC-MNs is creating culture conditions that could replicate the intricate processes guiding MN differentiation and maturation in vitro, maximizing relevance to in vivo models or clinical samples (Sepehrimanesh and Ding, 2020; Ding, 2021). Assembling and optimizing culture systems is a prerequisite for obtaining high-quality iPSC-MNs and producing reliable results in disease modeling, drug screening, or cell therapy.

Under physiological conditions in the mammalian central nervous system (CNS), astrocytes play a pivotal role in supporting neuron survival and function though the secretion of trophic factors such as brain-derived neurotrophic factor (BDNF) and glial cell-derived neurotrophic factor (GDNF), as well as fostering a conducive microenvironment via interactions with the extracellular matrix and neighboring cells (Fang et al., 2019; Enright et al., 2020; Akter et al., 2021; Chiareli et al., 2021; Batenburg et al., 2023). Thus, the coculture of iPSC-MNs with astrocytes has gained prominence as the favored approach for sustained neural cell culture. Significantly, the synergistic interplay between MNs and astrocytes in coculture creates a more physiologically relevant and neuroprotective environment (Bouvier et al., 2022; Roqué et al., 2023), ultimately leading to higher cell survival rates and valuable insights for research in neurobiology and neurodegenerative diseases.

Despite these benefits, coculturing MNs and astrocytes presents challenges for biochemical investigations due to the coexistence of astrocytes in MN cultures, heterogeneous cell populations, and complicating the isolation of highly pure MNs, large MN clusters, and MN detachment during long-term culture (Gottschling et al., 2016; Qiu et al., 2018; Sepehrimanesh et al., 2021; Castellanos-Montiel et al., 2023; Holt, 2023; Leng et al., 2023). Additionally, the inherent heterogeneity, altered gene expression patterns, and technical challenges associated with coculture systems can hinder the precision and accuracy required for robust scientific findings. This underscores the critical need to develop enhanced culture conditions for hiPSC-MNs to overcome these limitations and facilitate long-term studies (Roqué et al., 2023).

In this study, we established an indirect coculture system using culture inserts that physically separate iPSC-MNs and astrocytes. This method enabled us to achieve high yield and purity of iPSC-MNs at late maturation stages, making them suitable for biochemical studies. Leveraging this indirect coculture system, we successfully prepared highly pure MNs at late maturation stages from hiPSCs and conducted transcriptomic studies. Our analysis revealed genome-wide changes in gene expression in mature MNs, uncovering numerous genes associated with neurodevelopment and synapse organization and function. These findings highlight the high value of this coculture system in preparing hiPSC-MNs.

## Materials and Methods

### Cell lines, plasmids, and culture medium

HEK 293T cells (CRL-11 268) were purchased from ATCC. Human wild-type (WT) hiPSC (WTC11, UCSFi001-A) was obtained from the WiCell Research Institute. A third-generation lentiviral vector (pCSC-SP-PW-IRES-GFP) was used to express reprogramming factors NEUROG2-IRES-ISL1-T2A-LHX3 as described previously (Sepehrimanesh and Ding, 2020). The lentiviral vector was cotransfected with packaging plasmids (pCMV-Gag-Pol and pCMV-VSVG) into HEK293T cells for lentivirus production. Replication-incompetent lentiviruses were produced, and viral supernatants were collected at 48 and 72 h post transfection as previously described (Ding and Kilpatrick, 2013). The viral supernatants were filtered through 0.45 mm syringe filters and stored at 4°C before cell transduction. The medium recipes were as follows:

1. HEK medium: DMEM supplemented with 10% fetal bovine serum (FBS) and 1% penicillin/streptomycin (P/S).
2. Astrocyte medium: DMEM supplemented with 15% FBS and 1% P/S.
3. hiPSC medium: mTeSR1 Basal media (STEMCELL Technologies) supplemented with 5× supplement (STEMCELL Technologies) and 1% P/S.
4. KnockOut Serum Replacement (KOSR) medium: KOSR (Thermo Fisher Scientific), 1% GlutaMAX (Invitrogen) supplemented with 1% nonessential amino acid (NEAA; Invitrogen), 50 μM β-mercaptoethanol (β-ME; Invitrogen), and 1% P/S.
5. Neurosphere medium (NSP medium): DMEM/F12 medium containing 1% N2, 1% GlutaMAX, 1% NEAA, 50 mM b-ME, 1% P/S, 8 mg/ml Heparin, 20 ng/ml bFGF, and 20 ng/ml epidermal growth factor (EGF; PeproTech).
6. Neural progenitor cell medium: DMEM/F12 and neurobasal medium (1:1) containing 0.5% N2 (Invitrogen), 1% B27 (Invitrogen), 1% GlutaMAX, 1% NEAA, 50 mM b -ME, 1% P/S, 10 ng/ml EGF, and 10 ng/ml Basic fibroblast growth factor (bFGF).
7. Neuronal maturation medium: DMEM/F12 and neurobasal medium (1:1) containing 0.8% N2 (Invitrogen), 0.8% B27 (Invitrogen), 1% P/S, and supplemented with 5 mM FSK and 10 ng/ml each of BDNF, GDNF, and Neuotrophin-3 (NT3; PeproTech).

### Generation of hiPSC-MNs

MNs were prepared from hiPSCs as previously described (Sepehrimanesh and Ding, 2020; Ding et al., 2021; Akter et al., 2022). Briefly, hiPSCs were cultured in mTeSR1 medium with 10 µM all trans-retinoic acid (RA, Sigma) and 0.5 mM valproic acid (VPA) in Matrigel-coated 6-well plates for 7 d. Cells were then digested with Versene and gently resuspended as small aggregates in KOSR medium with 10 μM Y-27632 (STEMCELL Technologies). Cell clumps were cultured in the KOSR medium for 4 d, followed by the culture in the NSP medium for another week. The neurospheres were then dissociated into single cells with Accutase (Innovative Cell Technologies) and maintained in neural progenitor cell medium. For MN differentiation (Sepehrimanesh and Ding, 2020), neural progenitor cells were plated into Matrigel-coated plates at a density of 3 × 10^4^ cells/cm^2^ and transduced with a lentivirus expressing NEUROG2-IRES-ISL1-T2A-LHX3 (Sepehrimanesh and Ding, 2020). Culture medium was replaced the next day with neuronal maturation medium. Neurons were dissociated with Accutase on Day 5 and replated onto Matrigel-coated coverslips with or without the presence of astrocytes or in a culture insert depending on desired experiments. The neuronal maturation medium was half changed twice a week until analysis.

### Preparation of monolayer of astrocytes

Primary astrocytes were isolated from the cerebral cortices of postnatal days (P) 1–3 in either sex of mouse pups and subsequently cultured in DMEM supplemented with 15% FBS and 1% P/S. To prevent contamination, it is essential to conduct mouse dissection and brain culture procedures under aseptic conditions. All equipment, including the biosafety cabinet, forceps, scissors, and plates, should be sterilized before use. To initiate the process, ensure that 10 ml of 1× HBSS is prepared in a 15 ml Falcon tube and arrange the necessary dissection tools, a microscope, a cold light source, and 70% ethanol within the dissection hood. Begin by decapitating the mice, carefully removing the skin, and delicately extracting the skull using small scissors. Subsequently, use fine forceps to eliminate the olfactory bulbs and meninges carefully (Schildge et al., 2013; Guler et al., 2021). Once you have obtained meninges-free brains, rinse them thoroughly with HBSS. Add a small amount of astrocyte media to create a cell suspension and gently triturate the tissue. Gradually add more medium as needed. Finally, resuspend the cell pellets in a 10 cm culture plate precoated with gelatin and incubate them at 37°C with 5% CO_2_ in an incubator.

### Replating MNs into culture inserts

When astrocyte monolayer reaches ∼80–90% confluence within wells of 6-well culture plates, carefully position sterile culture inserts onto wells containing the astrocytes, ensuring a secure fit and proper alignment. Utilize Accutase to gently detach the MNs from culture dish, followed by neutralizing the Accutase with neuron maturation media to safeguard the MNs. Transfer the MNs suspension into a centrifuge tube and spin it (200 × *g*, 4 min) to form a pellet, carefully discarding the supernatant. Gently resuspend the MNs in neuron maturation medium and transfer the resuspended MNs onto the culture insert within the 6-well plate, which already contains the astrocyte monolayer. Adjust the MN density by adding an appropriate volume of the suspension. Finally, place the 6-well culture plate, with the insert and MNs, into a cell culture incubator with the required conditions for MN growth and maturation.

### Quantitative real-time PCR analysis

After culturing cells in the culture insert, the culture inserts were dismounted from the device and rinsed once with cold PBS. Total RNA was extracted from cultured cells using TRIzol (Life Technologies), and genomic contamination was removed using TURBO DNase (Life Technologies). cDNA synthesis reactions were performed using 0.5 µg of RNA from each sample with the SuperScriptIII First-Strand kit (Life Technologies) and random hexamer primers. Real-time PCR was performed in triplicate using primers, SYBR Green SuperMix (Invitrogen), and the BIO-RAD CFX-96 Fast Real-Time PCR system. Target mRNA levels were normalized to the reference gene GAPDH by 2-ΔΔCt method as described previously (Ding and Kilpatrick, 2013; Ding et al., 2016, 2018). The sequences of RT-PCR primers are as follows:

5′-GCACCAGTTCAAGCTCAAC-3′ (HB9-F)
5′-GCTGCGTTTCCATTTCATCC-3′ (HB9-R)
5′-CACCCAGCAGATGTTCGATG-3′ (TUBB3-F)
5′-CTGTTCTTGCTCTGGATGGC-3′ (TUBB3-R),
5′-ACTCCTGGAACCCCTAGCTA-3′ (MAP2-F)
5′-TGGGAGTCGCAGGAGATTTT-3′ (MAP2-R).

### RNA sequencing and bioinformatic analyses

The total RNA was extracted from cultured cells and purified using PureLink RNA Mini Kit (Invitrogen) according to manufacturer's instructions. The RNA integrity was determined by Agilent 2100 BioAnalyzer (Agilent Technologies) and sequenced using the Illumina NovaSeq at Novogene. The data obtained in FASTQ file format from RNA sequencing was aligned to Ensembl hg38 human genome using the HISAT2 program. Gene level abundances were estimated as FPKMs (fragments per kilobase of transcript sequence per millions base pairs sequenced). The read count summarization to each gene (i.e., counts) was calculated using featureCounts. Analyses of differential expression of transcripts were performed with DESeq2 (1.20.0). Genes with a false discovery rate (FDR) value of <1% and log_2_ fold-change ≥1 were considered to be differentially expressed genes (DEGs). The resulting *p* values of differential expression were accompanied with respective fold change values. The *p* values were adjusted for multiple testing by calculating FDR by Benjamini and Hochberg's method. Volcano plots and heat maps were created using the Rgplot and ggplot2 in Rstudio v1.1.463 (Anders and Huber, 2010; Walter et al., 2015). Fold changes (FC) >1.5 and *p* value <0.05 were considered the cutoff values for identifying upregulated and downregulated DEGs. The Database for Annotation, Visualization, and Integrated Discovery (DAVID, https://david.ncifcrf.gov/) was employed to perform gene ontology enrichment and pathway analysis. We submitted our lists of upregulated and downregulated genes into DAVID. A *p* value <0.05 was regarded as statistically significant, and the GO results were ranked by *p* value. The significant terms for Biological Processes (BP), Molecular Function (MF), and Cellular Component (CC; Mahmoodi et al., 2021) were selected. The significant terms for KEGG (Kyoto Encyclopedia of Genes and Genomes) and Reactome were selected.

### Immunocytochemistry

Cells on transparent culture insert were fixed with 4% paraformaldehyde (PFA) in 1× PBS for 15 min at room temperature in designated time point. After double washing with 1× PBS, cells were permeabilized and blocked for 1 h in a solution containing 1× PBS, 0.2% Triton X-100, and 3% BSA. Primary antibodies (Rabbit anti-TUBB3 (BioLegend; 1:2,000); Mouse anti-HB9 (DSHB; 1:500); Mouse anti-GFAP (Santa Cruz Biotechnology; 1:500) were added to the blocking solution and incubated overnight at 4°C. After three washes with PBS for 5 min each, coverslips were incubated with corresponding Alexa Fluor-conjugated secondary antibodies Donkey Anti-Rabbit IgG [H + L; Jackson ImmunoResearch; Alexa Fluor 594 (1:500)]; Donkey Anti-Mouse IgG [H + L; Jackson ImmunoResearch; Alexa Fluor 488 (1:500)] for 2 h at room temperature. Coverslips were then washed twice with PBS for 5 min each, and cell nuclei were stained with Hoechst 33342 (HST, Thermo Fisher Scientific). After an additional 5 min washing step with 1× PBS, the coverslips were mounted onto microscopy slides using Antifade Mounting Medium (Vector Laboratories).

### Survival assay

Induced neurons were replated onto 96-well plates coated with astrocytes, with a cell density of 1 × 10^3^ cells/cm^2^ and were subsequently maintained in a neuronal maturation medium. The culture medium was refreshed biweekly until the desired time points for analysis via immunostaining. A surviving neuron was defined by the characteristic neuronal morphology using TUBB3 immunostaining and contains a clear nucleus stained with HST. The number of neurons at the first week postviral infection (wpi) was set as 100% and served as the basis for normalizing the counts of surviving neurons at 2, 3, and 4 wpi. A degenerated neuron will be identified by the detachment from the culture plate and become floating in the culture medium. The number of floating neurons in a culture plate was estimated to evaluate the percentage of degenerated neurons. For each time point, three wells of neurons were examined under each condition.

### Electrophysiology

To confirm the electrophysiological maturation of neurons, whole-cell patch-clamp recordings were employed, following a methodology similar to previous studies (Sepehrimanesh and Ding, 2020; Ding et al., 2021) with minor adjustments. Specifically, induced MNs were cultivated on astrocyte-coated glass coverslips for 3 wpi before being subjected to analysis. These cells were carefully maintained at a constant temperature of 30°C within a submersion chamber, utilizing a Tyrode’s solution composed of (in mM) 150 NaCl, 4 KCl, 2 MgCl_2_, 3 CaCl_2_, 10 glucose, and 10 HEPES at pH of 7.4 (adjusted with KOH), and having an osmolarity of 300 mOsm. The recording pipettes, with resistances measuring ∼6–9 MΩ, were filled with an intracellular solution containing the following (in mM): 0.2 EGTA, 130 K-gluconate, 6 KCl, 3 NaCl, 10 HEPES, 4 ATP-Mg, 0.4 GTP-Na, and 14 phosphocreatine-di (Tris) at a pH of 7.2 and an osmolarity of 285 mOsm.

To record action potentials (APs), the cells were placed in current-clamp mode and stimulated using a series of current injections ranging from −20 to 200 pA at 20 pA increments, each lasting 800 ms. These current-clamp recordings were conducted either at the resting membrane potential or without any concurrent current injection. Subsequent data analysis was conducted utilizing Clampfit 10.3 software by Molecular Devices. For AP analysis, the AP trace situated just above the threshold was employed to determine the delay of the first spike, measuring the time elapsed from the initiation of the current steps to the peak of the AP. Furthermore, the same AP trace was utilized to gauge the AP threshold, pinpointing the voltage level corresponding to the steepest change in trace slope. Finally, the aforementioned AP trace was also scrutinized to ascertain the maximum velocity of both its ascent and descent. The frequency of APs was computed by dividing the maximum number of spikes observed during the current step protocol by the duration of each step, which was set at 800 ms.

### Imaging and quantification

Living cells were visualized in culture plates with a CKX53 inverted microscope (Olympus). Immunostaining images were obtained with a Leica (TCS-SP5) confocal microscope. An hiPSC-induced neuron was defined by highly expressed neuron-specific markers TUBB3 and MAP2. An induced MN was determined by the robust expression of nuclear markers, HB9 and ISL1, at early to mature stages. MNs are also immunostained with ChAT at 3 wpi to show late-stage mature neurons. The yield and the surviving neurons were counted based on TUBB3 signals and normalized to the number of starting materials.

### Statistical analysis

Statistical analysis was done in GraphPad Prism. The D’Agostino and Pearson omnibus normality test was conducted first to determine if the data are normally distributed. If the data passed the normality test, one-way or two-way ANOVA was used to determine significance. If the data did not pass the normality test, the Kruskal–Wallis test was used to determine significance. Results are expressed as mean ± SEM of at least three biological replicates, and *p* < 0.05 is considered significant.

## Results

### Limited survival observed when culturing hiPSC-MNs alone

**Figure 1.**
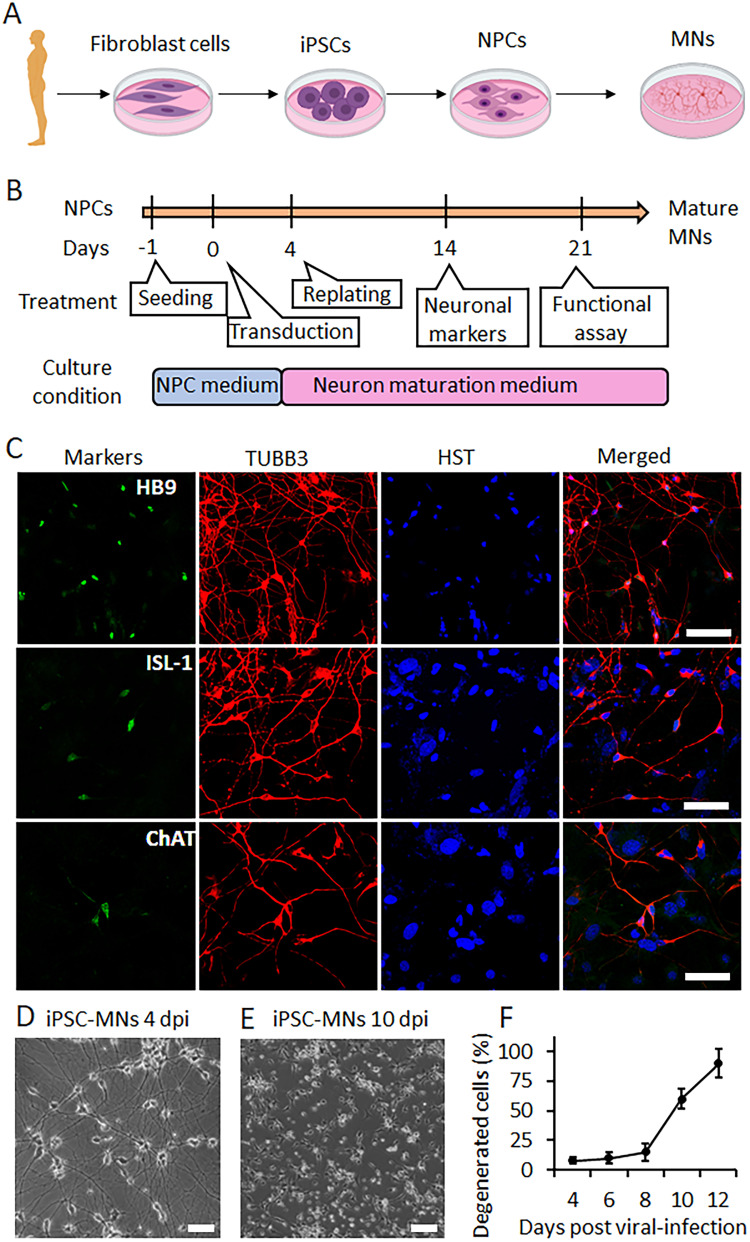
Limited survival when culturing iPSC-derived MNs alone. ***A***, Schematic shows the process of generation iPSC-MNs. iPSC, induced pluripotent stem cells; NPCs, neuronal progenitor cells; MNs, motor neurons. ***B***, The timeline, treatment, and culture conditions during the process of generation of iPSC-MNs. The time at lentiviral transduction was set as Day 0. ***C***, Representative confocal images of iPSC-MNs directly cocultured with astrocytes at 14 d postviral infection (dpi; for HB9 and ISL1) and 21 dpi (for ChAT). TUBB3 (tubulin beta 3 class III) served as a neuronal marker and Hoechst 33342 (HST) stained nuclei. Nuclear HB9 (motor neurons and pancreas homeobox 1) and ISL1 (ISL LIM homeobox 1) were used as early-stage MN markers, and ChAT (choline acetyltransferase) was used as a late-stage MN marker. Scale bar, 25 μm. ***D***, Representative micrograph of iPSC-MNs cultured alone at 4 dpi shows typical healthy outgrowth with complex neurites that are well attached on the culture plate. Scale bar, 100 μm. ***E***, Representative micrograph of iPSC-MNs cultured alone at 10 dpi shows most neurons are degenerated and start floating. Scale bar, 100 μm. ***F***, Percentage of neurons showed degeneration at different time points. Results represent three independent experiments. Data presented as mean ± SD.

The generation of hiPSC-MNs involves a sequential series of steps. This process encompasses hiPSC induction, the formation of embryoid bodies, the growth of neural rosettes, the differentiation of neuron progenitor cells (NPCs), transduction of lentiviral vectors expressing transcription factors, and finally, the induction and maturation of MNs (Sepehrimanesh and Ding, 2020; Akter et al., 2021, 2022; Fig. 1*A*). Throughout these processes, distinct culture media supplemented with specific factors are employed to nurture and guide the cells toward the MN lineage. The cells exhibit distinctive growth patterns during each differentiation phase, manifest unique cellular morphologies, and express key identity markers (Fig. 1*B*). These features are valuable in confirming cell identity at each induction stage and conducting quality assessments to obtain highly pure MNs.

Postmitotic MNs can be identified by specific MN markers, such as nuclear ISL LIM homeobox 1 (ISL1) and homeobox HB9 (HB9), while more mature MNs express choline acetyltransferase (ChAT; Fig. 1*C*; Arber et al., 1999; Karumbayaram et al., 2009; Amoroso et al., 2013; Kuijlaars et al., 2016). At 4 d postviral infection (dpi) of NPCs, induced MNs exhibit typical MN morphology with exceptionally long exons (Fig. 1*D*). When cultured alone, these MNs undergo gradual degeneration after 8 dpi, with most cells becoming detached and dying by 12 dpi (Fig. 1*E*,*F*). Despite the inclusion of advantageous growth factors such as BDNF, GDNF, and NT3, in specialized neuronal maturation culture media, the viability of MNs remains limited. This limited viability under this culture system poses a significant hurdle for modeling neurological diseases with mature hiPSC-MNs, which require >20 dpi to reach fully functional maturation (Ding, 2021).

### Coculturing with astrocytes enables full maturation of hiPSC-MNs

**Figure 2.**
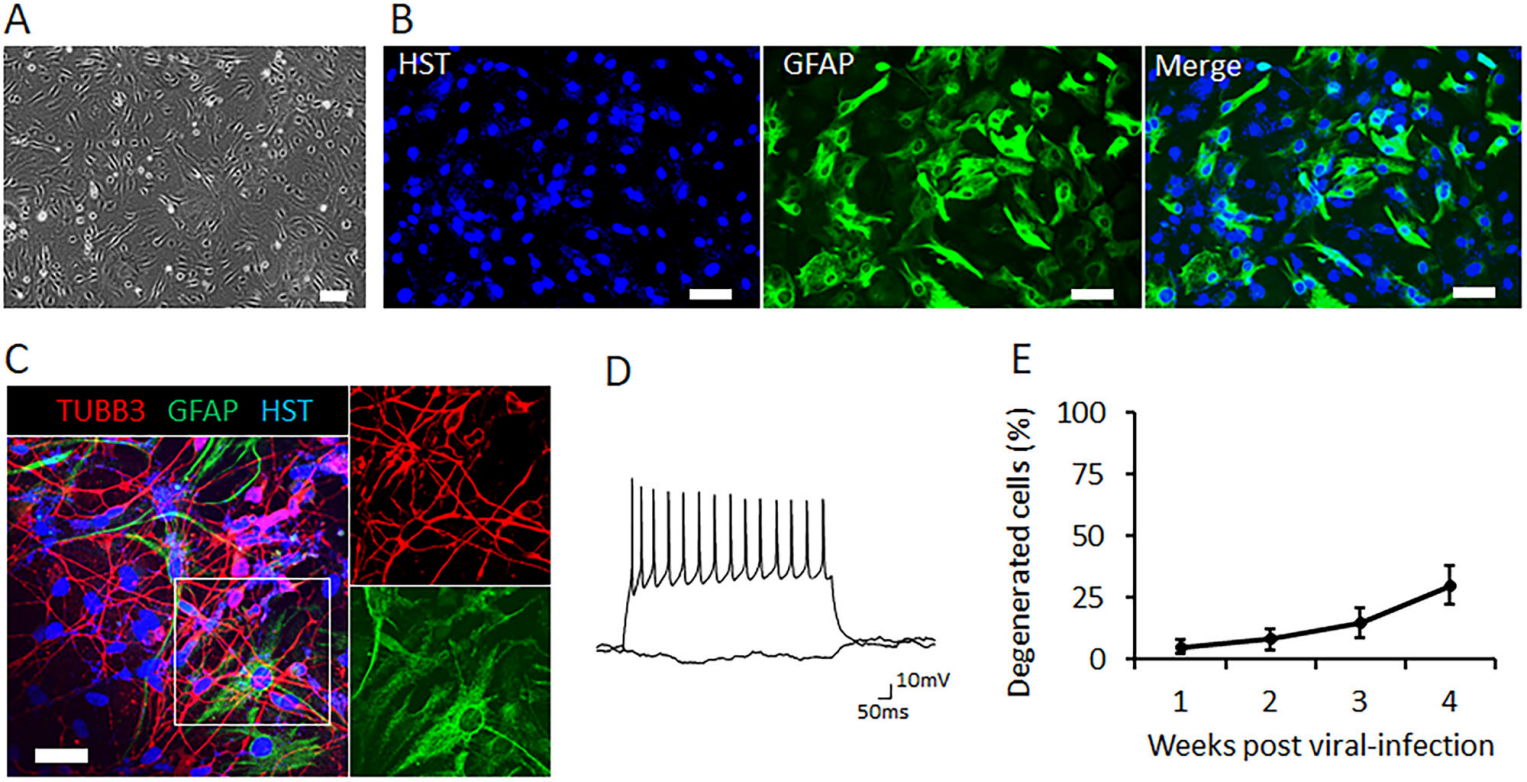
Coculturing with astrocytes enables full maturation of iPSC-MNs. ***A***, A representative micrograph of mouse primary astrocytes. Scale bar, 50 μm. ***B***, Confocal micrographs of primary astrocytes immunoassayed with astrocyte marker, glial fibrillary acidic protein (GFAP), and the nuclear dye Hoechst 33342 (HST). Scale bar, 25 μm. ***C***, Confocal micrograph of iPSC-MNs directly cocultured with astrocytes at 3 weeks postviral infection (wpi). Scale bar, 25 μm. ***D***, Repetitive AP waveforms recorded under current-clamp mode of iPSC-MNs at 3 wpi. ***E***, Degenerated neurons at different time points. Data presented as mean ± SD.

Under physiological conditions in the human brain, glial cells such as astrocytes are crucial for supporting neuron survival and functions (Pathak and Sriram, 2023). Coculturing neuronal cells with astrocytes has been demonstrated to help alleviate survival limitations (Kaech and Banker, 2006; Fath et al., 2009; Kerkering et al., 2023). To optimize the culture system and assess the long-term survival of cultured hiPSC-MNs, we established a direct coculture system where hiPSC-MNs were directly seeded onto the monolayer of astrocytes in culture plates. Mouse primary astrocytes were isolated from newborn pups and replated onto Matrigel-coated coverslips (Fig. 2*A*). Most cells robustly expressed specific astrocyte markers, such as glial fibrillary acidic protein (GFAP; Fig. 2*B*), confirming the purity and cell identity of purified astrocytes. These cocultured neurons exhibited healthy morphological integrity, as indicated by immunostaining of the generic neuron marker, tubulin beta 3 class III (TUBB3; Fig. 2*C*). At a late mature stage of 3 weeks postviral infection (wpi), patch-clamp recordings demonstrated that these neurons fired repetitive APs on the current injection (Fig. 2*D*). The electrophysiological activity of mature MN reflects their ability to form functional neuronal network connections. In comparison with MNs cultured alone, where all neurons died at 12 dpi (Fig. 1*F*), >50% of neurons cocultured with astrocytes remained viable without noticeable degeneration at 4 weeks (Fig. 2*E*), suggesting a dramatic enhancement in hiPSC-MN survival under coculture conditions. Thus, coculturing with astrocytes is both necessary and sufficient to support the long-term culture of hiPSC-MNs and achieve full maturation.

### Assembling a coculture system using culture inserts to physically separate astrocytes and iPSC-MNs

**Figure 3.**
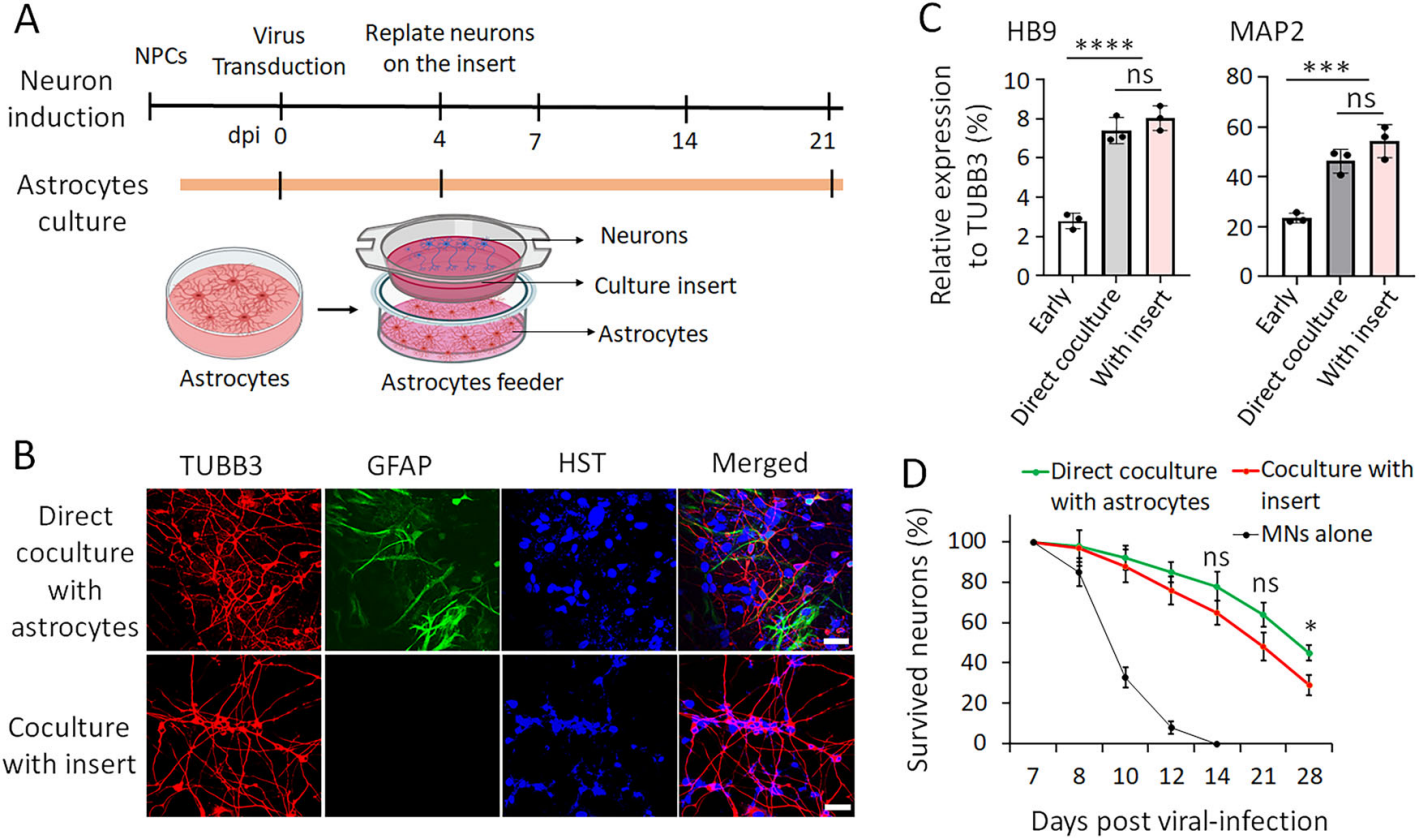
Assembling a coculture system using culture inserts to physically separate astrocytes and MNs. ***A***, Schematic shows the process of assembling the coculture system with culture inserts that physically separate neurons and cocultured astrocytes. ***B***, Representative confocal micrographs of MNs at 14 dpi with direct coculture with astrocytes and coculture with astrocytes using culture insert. Neuronal marker TUBB3 shows the soma and neuron processes, Hoechst 33342 (HST) stained nuclei, and glial fibrillary acidic protein (GFAP) was used as an astrocyte marker. Scale bar, 25 μm. ***C***, RT-PCR assay shows the relative gene expression levels of HB9 and MAP2 in indicated conditions. Samples of iPSC-MNs at 7 dpi were set up as an early-stage control, and samples of cocultured iPSC-MNs with culture inserts were collected at 21 dpi. Target gene expression was normalized with TUBB3. Data presented as mean ± SD. ns, not significant; ***p < 0.001; ****p < 0.0001. ANOVA. ***D***, The time course of survived neurons under indicated conditions. The number of neurons at 7 dpi was set as a starting number (100%) and the numbers of surviving neurons at late time points were normalized by the starting number. Results represent three independent experiments. Data presented as mean ± SD. Direct coculture versus coculture with inserts: ns, not significant; *p < 0.05. Student's *t* test.

The direct coculture of hiPSC-MNs with astrocytes significantly improves survival and provides excellent materials for immunocytochemistry-based analysis, through which neurons can be identified from astrocytes using specific markers. However, this direct coculture system limits its application in biochemical studies, which usually require highly pure neurons. We are wondering whether a similar survival support could be achieved in an indirect coculture system, in which astrocytes and MNs are physically separated. To this end, we used cell culture inserts to physically separate astrocytes and MNs through a semipermeable membrane in a transwell system (Fig. 3*A*). We cultured astrocytes in the culture wells, allowing them to form a monolayer with ∼80–90% confluence. Subsequently, we positioned the culture insert into the well of culture plate and replated MNs onto the culture inserts at an early stage of 4 dpi. The porous membrane of the culture inserts is designed for versatility, allowing MNs to be simultaneously exposed to growth media and astrocyte-secreted factors. We have closely examined and compared hiPSC-MNs prepared using this indirect coculture system with the conventional direct coculture method. The hiPSC-MNs cultured in culture inserts showed healthy growth with typical neuronal morphology as MNs directly cocultured with astrocytes (Fig. 3*B*).

To further characterize the neurodevelopment of iPSC-MNs prepared with different coculture methods, we collected RNA samples at various developmental stages and performed a quantitative polymerase chain reaction (qPCR) assay. Samples at 7 dpi served as an early developmental stage control, while samples at 21 dpi represented the maturation stage (Ding et al., 2021). After normalization with the reference gene of the generic neuron marker TUBB3, the expression levels of MN marker HB9 and the developmental marker MAP2 significantly increased at 21 dpi compared with 7 dpi (Fig. 3*C*), suggesting that induced neurons acquired MN identity and reached maturation. Notably, no significant differences were detected in the gene expression of HB9 and MAP2 in MNs prepared using these two coculture systems (Fig. 3*C*), indicating that cell culture insert does not significantly influence the expression of critical genes during maturation.

Further investigation focused on the time course of neuronal survival under different conditions: iPSC-MNs cultured alone, directly cocultured with astrocytes, and cocultured with culture inserts. Neuronal survival was assessed relative to the starting number of neurons at 7 dpi, which was set as 100%. Consistently (Fig. 1*F*), MNs cultured alone exhibited degeneration after 8 dpi and cannot survive beyond 14 dpi. In contrast, both direct coculture and coculture with inserts demonstrated similar levels of neuronal survivability over time (Fig. 3*D*), indicating the robust protective effect of cocultured astrocytes on iPSC-MNs. Importantly, statistical analysis revealed no significant difference in neuronal survival between these two coculture conditions within 3 wpi, with only slightly fewer surviving neurons in the culture insert condition than the direct coculture at 4 wpi (Fig. 3*D*). This result indicates that the indirect coculture system possesses a similar protective effect on neuronal survival. Together, direct coculture with astrocytes significantly enhances iPSC-MN survival, and the indirect coculture system using culture inserts can achieve the similar protective effect on neuronal growth, development, maturation, and long-term survival in cultures.

### iPSC-MNs prepared using culture inserts exhibit a typical neurodevelopmental switch in gene expression during maturation

**Figure 4.**
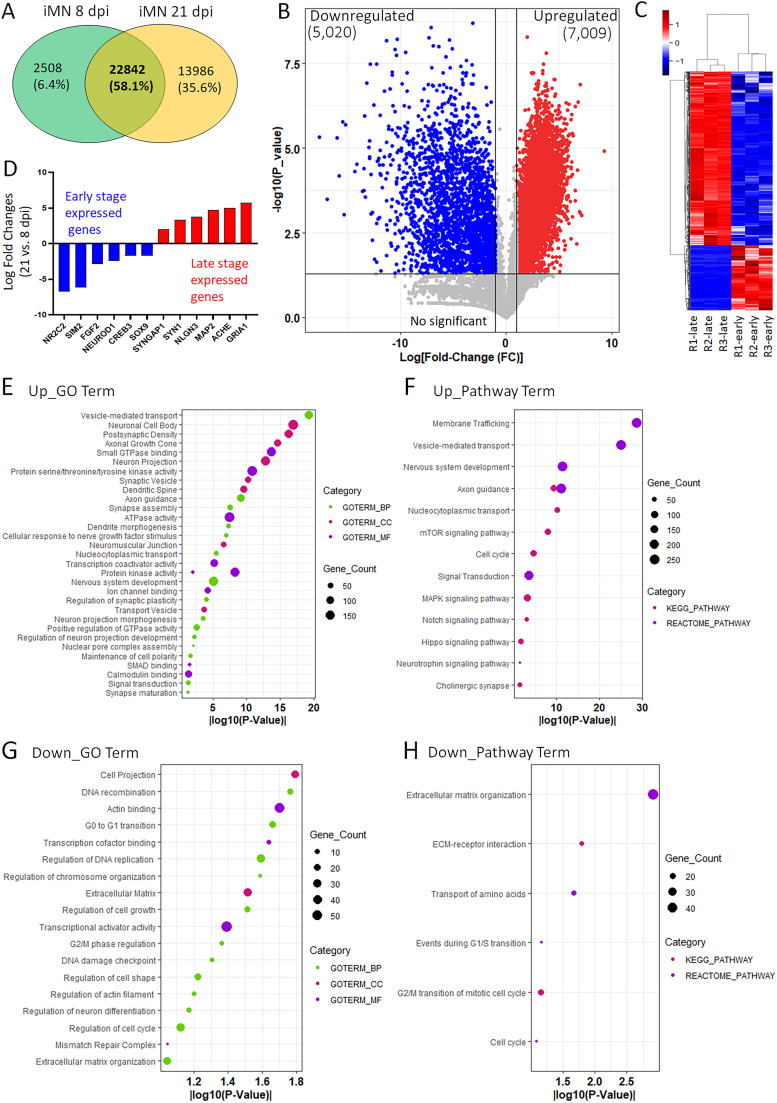
Transcriptomic studies characterized iPSC-MNs prepared using culture inserts. ***A***, Venn diagram shows the RNAseq results of iPSC-MNs at different development stages. The early-stage samples of iPSC-MNs (8 dpi) were cultured alone and the late-stage samples of iPSC-MNs (21 dpi) were cultured using culture inserts. ***B***, A volcano plot shows the DEGs (21 vs 8 dpi) based on the cut off log_2_FC >1 and *p* value <0.05. Blue and red dots represent down- and upregulated genes, respectively. Gray dots represent the remaining genes with no significant difference. ***C***, Heat map of RNAseq results of all DEGs in iPSC-MNs (21 vs 8 dpi), including 5,020 downregulated genes and 7,009 upregulated genes. ***D***, The developmental switch of some temporally regulated genes in iPSC-MNs at 21 dpi. Genes highly expressed at early developmental stages were downregulated, and genes implicated in neuron maturation are upregulated. ***E***, GO analysis of significantly upregulated genes in iPSC-MNs at 21 dpi with the enrichment of terms related to neuron maturation and synaptogenesis. ***F***, Pathway enrichment analysis with KEGG and Reactome of significantly upregulated genes in iPSC-MNs at 21 dpi. ***G***, GO analysis of significantly downregulated genes in iPSC-MNs at 21 dpi with the enrichment of GO terms related to early neuron differentiation. ***H***, Pathway enrichment analysis with KEGG and Reactome of significantly downregulated genes in iPSC-MNs at 21 dpi.

To further characterize the neurodevelopment and maturation of iPSC-MNs prepared using cell culture inserts, we conducted transcriptomic studies to understand the genome-wide changes in gene expression during the process from early differentiation to late maturation. Highly pure MNs were prepared from a healthy iPSC line at an early differentiation stage of 8 dpi and at a late maturation stage at 21 dpi. Bioinformatic analysis identified 12,029 DEGs among 22,842 hits, consisting of 5,020 downregulated genes and 7,009 upregulated genes in MNs at 21 dpi compared with 8 dpi (Fig. 4*A–C*). Consistent with known knowledge regarding neurodevelopment (Ding et al., 2013; Ding, 2015), a significant developmental switch in gene expression can be observed, with genes highly expressed at early development stages being downregulated, while genes associated with late neuron maturation are upregulated (Fig. 4*D*). Many downregulated genes are transcription factors or regulators participating in cell amplification and early neuron differentiation. For example, the *NR2C2* gene, encoding the Nuclear Receptor Subfamily 2 Group C Member 2, a key regulator of genes associated with stem cell self-renewal, cell commitment, and neuronal differentiation (Brockington et al., 2010). The *SIM2* gene, encoding a member of the basic Helix-Loop-Helix/PER-ARNT-SIM (bHLH/PAS) family of transcription factors, is expressed within the brain and play important roles in brain development and function (Button et al., 2022). Another transcription factor SOX9 also exhibited a significant decrease in late stage iPSC-MNs (Fig. 4*D*), consistent with previous reports that SOX9 robustly expressed in neural stem cells but decrease during distinct stages of neuronal differentiation (Stolt et al., 2003; Nagakura et al., 2020).

On the other hand, genes upregulated in late-stage iPSC-MNs are closely related to neuronal maturation and function, with enrichment in synaptogenesis. For example, the *GRIA1* gene encodes the glutamate ionotropic receptor AMPA type subunit 1 (Shen and Limon, 2021). The *MAP2* gene encodes the microtubule-associated protein that determines the dendritogenesis during neuron development (DeGiosio et al., 2022). The *SYNGAP1* gene encodes a Ras GTPase activating protein and plays critical roles in regulation of synaptic plasticity and neuronal homeostasis (Mignot et al., 2016). Other upregulated genes’ products, such as *SYN1*, *NLGN3*, and *ACHE*, either directly involve synaptogenesis or neurotransmitter metabolism. This shift in gene expression patterns reflects the specialization and maturation of hiPSC-MNs transitioning from early differentiation to late maturation (Fig. 4*D*).

The DEGs, both upregulated and downregulated, represent a diverse array of intriguing targets. Utilizing DAVID Bioinformatics Resources (Huang et al., 2009), we conducted an in-depth analysis of these DEGs using Gene Ontology (GO) to unravel their molecular functions and biological processes. The GO analysis identified broad changes in upregulated DEGs primarily linked to vital biological processes related to neurodevelopment, including nervous system development, signal transduction regulating neuron projection development, axon guidance, dendrite morphogenesis, synapse assembly and maturation, maintenance of cell polarity, and nucleocytoplasmic transport (Fig. 4*E*). The products of these upregulated DEGs are widely distributed across various cellular components, with notable enrichment in membrane-bound organelles such as neuronal cell bodies, synapses, synaptic vesicles, postsynaptic density, and axonal growth cones (Fig. 4*E*). These upregulated DEGs are associated with diverse molecular functions, including ATPase and protein kinase activities, as well as binding activities with SMAD and calmodulin, relevant effectors such as channels, receptors, synaptic, and structural genes among others. The implicated KEGG and Reactome pathways encompass essential cellular processes such as the cell cycle, signal transduction, notch and hippo signaling pathways, neurotrophin signaling pathway, cholinergic synapse, and vesicle-mediated transportation (Fig. 4*F*).

Conversely, downregulated genes exhibit substantial enrichment in biological processes intricately linked to cell amplification, including the regulation of DNA replication, recombination, chromosomal organization, G0–G1 phase transition, G2/M phase regulation, and cell cycle (Fig. 4*G*). The associated cellular components involve cell projections and the extracellular matrix, while the molecular functions include transcription cofactor and activator activities. The implicated KEGG and Reactome pathways comprise crucial events in the cell cycle such as G2/M and G1/S transitions, as well as extracellular matrix organization, among others (Fig. 4*H*). Notably, this study represents the first comprehensive assessment of gene expression across the genome using highly pure iPSC-MNs at late maturation stage. Consistent with previous studies using primary neurons (Ding et al., 2013, 2016, 2018), this transcriptomic study illustrates that the multifunctionality of transcriptional regulators governs the temporal regulation of gene expression, one timing mechanism of gene expression in the process of neurodevelopment and maturation (Ding, 2015). Importantly, the transcriptomic data reveals a core set of DEGs from neuronal progenitor cells to mature iPSC-MNs, representing a maturation program that is also identified in in vivo systems (Shimojo et al., 2015; Ciceri et al., 2024).

### Characterization of gene expression in hiPSC-MNs at late maturation stages

**Figure 5.**
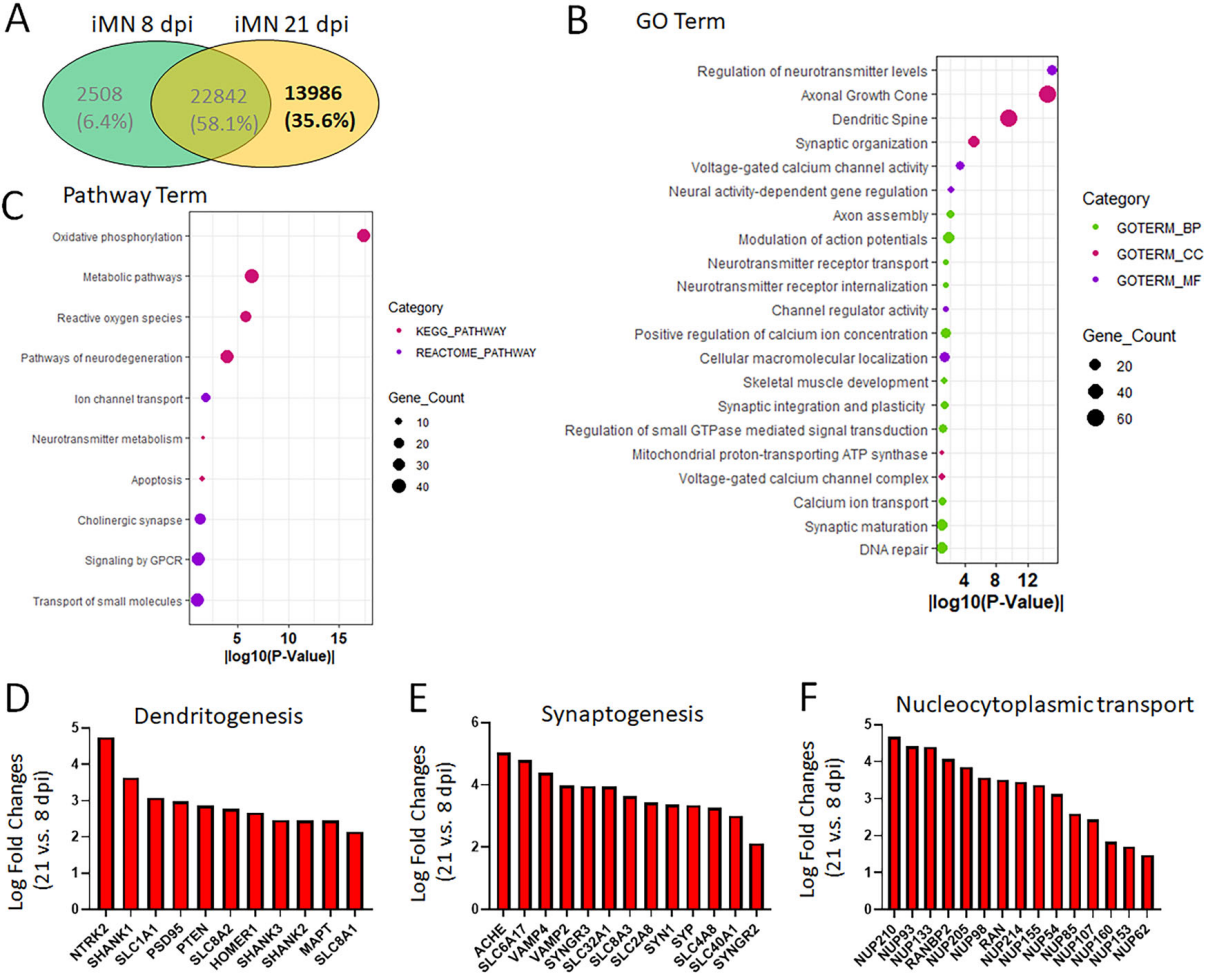
Characterization of gene expression in hiPSC-MNs at late mature stages. ***A***, Venn diagram shows the RNAseq results of the number of genes that were only identified in late-stage iPSC-MNs at 21 dpi prepared with culture inserts. ***B***, GO analysis of genes only expressed in iPSC-MNs at 21 dpi with the enrichment of terms related to neuron functions. ***C***, Pathway enrichment analysis with KEGG and Reactome of genes only expressed in iPSC-MNs at 21 dpi. ***D***, Representative genes involved in dendritogenesis are upregulated in iPSC-MNs at 21 dpi. ***E***, Representative genes involved in synaptogenesis are upregulated in iPSC-MNs at 21 dpi. ***F***, Representative genes involved in nucleocytoplasmic transport are upregulated in iPSC-MNs at 21 dpi.

The bioinformatic analysis of RNA sequencing data revealed 13,986 genes (constituting 35.6% of the total DEGs) exclusively expressed in hiPSC-MNs at the late maturation stage of 21 dpi compared with the early stage of 8 dpi (Fig. 5*A*). The products of these genes participate in various biological processes and play crucial roles in neuronal maturation and functions. For examples, they are involved in the regulation of neurotransmitter levels, neurotransmitter receptor transport and internalization, axon assembly and growth, modulation of APs, calcium ion concentration and transport, synaptic organization, maturation, integration, and plasticity. These genes expressed at the mature stage are also associated with voltage-gated calcium channels, calcium ion transport, and channel regulator activity (Fig. 5*B*). KEGG and Reactome pathway analysis identified that these genes are highly enriched in key pathways critical for the physiological functions of mature MNs, such as oxidative phosphorylation, metabolic processes, ion channel transport, neurotransmitter metabolism, cholinergic synapse, and signaling by G-protein-coupled receptors (Fig. 5*C*). The high expression of these late mature genes further demonstrates that iPSC-MNs prepared using culture inserts achieved fully functional maturation at 21 dpi.

Furthermore, we closely analyzed the most significantly upregulated genes at 21 dpi compared with 8 dpi and found that these genes are highly enriched in several groups, such as dendritogenesis, synaptogenesis, and nucleocytoplasmic transport, for example, the genes of *SHANK1*, *SHANK2*, and *SHANK3* (Fig. 5*D*), which encode Shank proteins functioning as scaffolding molecules in the postsynaptic density of neurons. The upregulation of these genes promotes dendrite outgrowth and maturation, dendritic spine enlargement especially in spine heads, spine morphogenesis, and synaptic activity (Hung et al., 2008; Unsicker et al., 2021). Synaptic connection markers (e.g., *SYN1* and *SYP*), along with other regulatory proteins for synaptic vesicles (e.g., *CLC6A17*, *SYNGR3*, and *SYNGR2*), and proteins controlling synaptic vesicle release (e.g., *VAMP2* and *VAMP4*), are robustly expressed in late-stage iPSC-MNs (Fig. 5*E*). These proteins participate in synaptic connectivity, dendritic arborization, and dendritic outgrowth and are extensively employed parameters for assessing neuronal maturation (Raingo et al., 2012; Alves et al., 2015; Lamotte et al., 2020; Simmons et al., 2020; Santoso et al., 2021; Patel et al., 2022; Petanjek et al., 2023).

Interestingly, another group of genes are significantly involved in nucleocytoplasmic transport, a biological process regulating bidirectional transport across the nuclear envelope (Ding and Sepehrimanesh, 2021). For example, genes encoding nucleoporins (NUPs) and the small GTPase RAN are highly expressed in iPSC-MNs at 21 dpi (Fig. 5*F*). NUPs are subunits for assembling nuclear pore complexes, which serve as the principle gateway for the nuclear transport (Ding et al., 2020; Coyne and Rothstein, 2022), while the RAN-GTPase together with its regulators are required for the generation and maintenance of the RAN gradient, which provides the driving force for cargos passing through the nuclear pore complex (Ding et al., 2020; Akter et al., 2024). The increased expression levels of NUPs and RAN in late-stage iPSC-MNs indicate the higher demand for nuclear transport activities in maturing neurons.

Taken together, our results demonstrated that coculture with astrocytes is necessary and sufficient to support the iPSC-MNs in reaching maturation stages. The indirect coculture system using cell culture inserts enables the obtaining of mature iPSC-MNs with comparable quality to the direct coculture system, providing a unique strategy for preparing highly pure iPSC-MNs at late mature stages for biochemical studies.

## Discussion

Pluripotent stem cell-derived neurons offer an unprecedented approach for disease modeling, drug screening, and cell therapy (Kimbrel and Lanza, 2020; Yamanaka, 2020). However, obtaining high-quality induced neurons with good yield and purity is a significant challenge in this research field. Firstly, the purity and neuronal identity of induced neurons vary among different studies using various induction approaches. For example, iPSC-MNs can be generated using lentiviral delivery of transcription factors or defined small molecules (Qu et al., 2014; Ben-Shushan et al., 2015; Sepehrimanesh and Ding, 2020; Akter et al., 2022). Although these iPSC-MNs express MN markers of nuclear HB9 and CHAT, significant differences can be observed in purity, morphology, and characterization during maturation between MNs prepared by different approaches (M. Sepehrimanesh et al., unpublished observations). Secondly, the limited survival of induced neurons is another major challenge. Despite the inclusion of various neurotrophic factors and supplements in the culture medium, highly pure iPSC-MNs cultured along only typically survive ∼10 d, falling short of reaching full maturation stages. We have observed that the higher the purity of induced neurons, the shorter their survival time when cultured alone. This survival issue significantly hampers the application of hiPSC-derived neurons in modeling age-related neurological diseases, wherein disease-dependent neuronal deficits typically manifest during maturation stages.

In this study, we comprehensively assessed the neuronal survival and maturation of iPSC-MNs under different culture conditions. We demonstrated that coculture with astrocytes is necessary and sufficient to support iPSC-MNs in achieving full maturation with typical MN morphology and characterization. Importantly, we also developed an indirect coculture system using culture inserts that physically separate astrocytes and iPSC-MNs. With this system, we obtained highly pure neurons at late mature stages, comparable in quality to direct coculture with astrocytes. This advancement makes it possible to biochemically identify pathogenic factors using diseased neurons at late disease onset stages. We believe that this indirect coculture system will be applicable for the preparation of various types of iPSC-derived neurons, providing a unique technique in this research field.

In this study, primary astrocytes isolated from newborn pups were used to coculture with iPSC-MNs. Interestingly, when we tested the coculture with some human astrocyte cell lines, we found that the neuronal survival was not as good as with primary mouse astrocytes. This difference could be due to the age and passage number of astrocytes. It would be interesting to set up the coculture with iPSC-derived astrocytes to see if neuronal survival improves, given that both astrocytes and neurons would be derived from the same iPSC line.

In the mammalian CNS, MNs originate from MN progenitor cells and undergo a successive process of development and maturation (Stifani, 2014). The interactions between environmental cues and the intrinsic developmental program, which regulates gene expression, govern the acquisition of the MN fate and the process of maturation (Novitch et al., 2003; Ding, 2015). Consistent with this established knowledge, our transcriptomic study revealed a clear developmental switch in gene expression in iPSC-MNs during the transition from the early immature stage to the late mature stage. In this developmental switch, transcription regulators involved in MN differentiation at the early developmental stage are downregulated, while genes participating in synaptogenesis and neuronal functions are upregulated. The expression changes of similar or the same gene sets have been reported in MN maturation in other in vivo model systems (Goulding, 2009; Moreno and Ribera, 2010; Stifani, 2014; Wang et al., 2018), further demonstrating the high quality and identity of iPSC-MNs prepared with this coculture system.

In this study, we demonstrate how various culture conditions influence specific outcomes and pave the way for future endeavors in coculturing MNs with other cell types like muscle or different neuronal subtypes. Unraveling the molecular and cellular cues driving MN differentiation from hiPSCs holds promise for uncovering innovative therapies for neurodevelopment and neurodegenerative diseases.

## Synthesis

Reviewing Editor: Eran Perlson, Tel Aviv University Decisions are customarily a result of the Reviewing Editor and the peer reviewers coming together and discussing their recommendations until a consensus is reached. When revisions are invited, a fact-based synthesis statement explaining their decision and outlining what is needed to prepare a revision will be listed below. The following reviewer(s) agreed to reveal their identity: Shinghua Ding.

This study investigates the survival and maturation of human induced pluripotent stem cell (hiPSC)-derived motor neurons (MNs) using two different coculture systems with primary mouse astrocytes: direct coculture, where neurons and astrocytes are grown together, and indirect coculture, where they are separated by culture inserts. Both systems significantly enhance neuron survival and allow neurons to reach late maturation stages within three weeks. The indirect coculture system is particularly effective, yielding highly pure MNs at late maturation stages. Transcriptomic analysis of these neurons reveals a neurodevelopmental switch in gene expression, with mature genes associated with neurodevelopment and synaptogenesis highly enriched at late stages. The study offers a novel tool for preparing highly pure hiPSC-derived neurons, crucial for studying neurological diseases, especially age-related neurodegeneration, at late disease onset stages.

To enhance the manuscript's clarity and impact, the authors should address the issues outlined below, including clarifying methodological details, adjusting data presentation, and considering the use of human astrocytes. Once these revisions are made, the manuscript will offer a significant contribution to the field of iPSC-MN research and the modeling of neurodegenerative diseases.

Major comments:

1. The sentence in the abstract 'Both systems significantly enhance neuron survival with no significant differences in neurodevelopment, maturation, and survival within 3 weeks, allowing them to reach maturation stages' is unclear. It says...enhance neuronal survival, then no significance in ...survival in 3 weeks

2. how to calculate the survival IMNs should be clarified in the survival assays. Based on the text, it should be calculated as Tubb3+cells/Total HST+ cells?

3. In Fig 1C, 20 dpi, the % of Chat+ cells seems low, is this because of degeneration?

4. How to quantify degenerating cells in Fig 1D needs to be clarified.

5. '...demonstrated spontaneous electrophysiological activity with repetitive action potentials (Figure 2D)' (line 300). Spontaneous activity is not visible, and may change the scale of current. I believe APs were induced by the current injection.

6. Please check the DEG number in Fig 4A, it is not consistent with the description in the text (line 354-358)

7. Heatmap of Fig 4C is about all DEGs or with genes associated with early and late neuronal maturation genes?

8. In line 372, 'On the other hand, genes temporally upregulated in late-stage iPSC-MNs are closely relayed to neuronal maturation and function...', temporally should be deleted as it is related to the maturation, and 'relayed' should be 'related'.

9. Since iPSCs and iMNs are derived from humans, it would be more significant to use human astrocytes (human astrocyte cell line is available). This can be discussed in the discussion section.
